# Genome‐Wide Assessment of DNA Methylation Fidelity Identifies Representative Ovarian Cancer Cell Line Models

**DOI:** 10.1111/jcmm.71334

**Published:** 2026-08-22

**Authors:** Asia Jordan, Abby Caffrey, Carson Stacy, Ruby Yun‐Ju Huang, Tuan Zea Tan, Aideen McCabe, Kellie Dean, Sudipto Das, Antoinette S. Perry

**Affiliations:** ^1^ School of Biology and Environmental Science, O'Brien Science Centre University College Dublin Dublin Ireland; ^2^ Cancer Biology and Therapeutics Laboratory UCD Conway Institute of Biomolecular and Biomedical Science, UCD Dublin Ireland; ^3^ School of Pharmacy and Biomedical Sciences Royal College of Surgeons Dublin Ireland; ^4^ School of Medicine, College of Medicine National Taiwan University Taipei Taiwan; ^5^ Cancer Science Institute of Singapore National University of Singapore Singapore Singapore; ^6^ School of Biochemistry and Cell Biology, Western Gateway 3.91 University College Cork Cork Ireland

**Keywords:** DNA methylation, histotype, ovarian cancer

## Abstract

Epithelial ovarian cancer comprises multiple distinct histotypes with different molecular, clinical, and therapeutic characteristics, yet ovarian cancer cell lines are often broadly classified as ‘ovarian carcinoma’ or ungraded ‘serous’. While studies have classified cell lines using mutations, transcriptomics, or immunophenotypes, their DNA methylation profiles remain poorly characterised. Given the role of DNA methylation in epithelial ovarian cancer, histotype‐specific methylation profiling is essential to determine how accurately cell lines model primary tumours and to guide their appropriate use. The Infinium 450 K and EPIC DNA methylation profiles of 60 unique ovarian cancer cell lines and 108 ovarian tumours were evaluated though a correlation and effect size analysis, focusing on genetic and epigenetic domains. DNA methylation patterns were found to be highly conserved between cell lines and tumour tissues at key transcriptional regulatory domains (CpG islands, shores, and 5′ regulatory regions), with these CpG‐dense loci more accurately reflecting in vivo conditions than CpG‐sparse regions. We identified specific cell lines with representative epigenetic signatures of high‐grade serous, clear cell, endometrioid, mucinous and mixed ovarian carcinoma histotypes. This study provides a framework to optimise the selection and application of ovarian cancer cell line models in DNA methylation research.

## 1 Introduction

Ovarian cancer (OvCa) has the highest mortality rate among all gynaecological malignancies. Despite decades of research, death rates have only marginally declined in some countries. Late‐stage diagnosis remains a challenge and therefore there is a critical need to develop improved treatment strategies alongside early detection technology [1].

Cell lines are a fundamental tool in understanding key biological mechanisms and testing therapeutic strategies in cancer. However, research utilising OvCa cell lines is impacted by the complexity of the disease, particularly its diverse range of histological subtypes, also known as histotypes. The most common form, epithelial ovarian carcinoma, was historically researched and treated as a single disease [2]. However, it is now understood that the disease encompasses a range of histotypes including high‐grade serous (HGSOC), low‐grade serous (LGSOC), clear cell (CCOC), endometrioid (EOC) and mucinous ovarian carcinoma (MOC) [3]. Each exhibits distinct molecular profiles, treatment responses, metastatic behaviours and prognoses [4, 5]. There are also less common epithelial OvCa histotypes, such as Brenner tumours, carcinosarcomas and undifferentiated carcinomas, while some patients present with mixed histologies (MXOC), meaning their tumours are comprised of two or more histotypes, further complicating diagnosis and treatment [6].

Overlooking these histotype distinctions in cell lines limits progress in diagnostics, prognostics and therapeutic development [7]. As most OvCa cell lines were developed when the disease was diagnosed and treated as a single entity, they are largely classified as ‘ovarian carcinoma’ or ungraded ‘serous’ (SOC) [8]; vague and overarching terms that fail to accurately distinguish their histotype origin. Insufficient histotype‐specific identification can lead to misuse and data misinterpretations, with experimental findings inaccurately reflecting the in vivo biology of specific histotypes.

Previous studies shed some light on the origin and histological subtype of OvCa cell lines through profiling DNA mutations, copy number alterations, transcriptomic analysis, immunological profiles and drug responses [8, 9, 10, 11, 12]. However, research into the epigenome of the different cell line histotypes is lacking. DNA methylation in particular has been shown to be both prognostic and predictive of survival and chemoresistance in HGSOC [13, 14, 15, 16]. DNA methylation alterations have also been used as therapeutic targets in CCOC [17] and as a predictive biomarker in MOC [18]. As such, classification of the cell line histotypes by DNA methylation profiling would support biomarker and therapeutic research. Cell lines remain indispensable experimental models for elucidating OvCa biology, assessing drug mechanisms of action and therapeutic efficacy. The translational relevance of findings derived from in vitro cell lines is contingent on the extent to which they simulate the molecular features of their tissues in vivo. Ensuring this representativeness is critical for generating reliable research outputs.

This aims of this study were to (1) determine whether DNA methylation profiles of OvCa cell lines are representative of their corresponding tissues and (2) identify histotypes of OvCa cell lines with unspecified origin, and lastly to (3) provide recommendations on which OvCa cell lines are best for DNA methylation studies.

## 2 Materials and Methods

### 2.1 Data Selection

Illumina Infinium DNA methylation EPIC and 450 K array datasets where obtained from the NCBI gene expression omnibus [19]. Three OvCa cell line datasets: GSE118405 (raw data provided by the Cancer Science Institute of Singapore) [20], GSE168225 [21], and GSE198073 [22] were used. These contained DNA methylation profiling data for 60 different OvCa cell lines (some with biological replicates) (Table S1). We assigned specific histologies to these cell lines using a combination of Cellosaurus (https://www.cellosaurus.org) [23], DepMap (https://depmap.org/portal) [24] and genomic, proteomic and molecular information from previous relevant literature [8, 9, 10, 11, 12] (Table S2). The assigned histologies of the cell lines used in this study are detailed in Table 1. The GSE168930 [25] dataset contained DNA methylation data for OvCa tissues: HGSOC (*n* = 38), SOC (*n* = 1), EOC (*n* = 20), CCOC (*n* = 25), MOC (*n* = 11), LGSOC (*n* = 1) and MXOC (*n* = 12). Grade 3 tumours were classed as high‐grade serous [26].

**TABLE 1:** Characterisation of DNA methylation data from ovarian cancer cell lines and tissues.

Category	Cell lines *n* (%)	Tissues *n* (%)
Total	130 (100)	108 (100)
Platform		
450 K array	116 (89)	0 (0)
EPIC array	14 (11)	108 (0)
Dataset		
GSE118405	116 (89)	0 (0)
GSE168225	4 (3)	0 (0)
GSE168930	0 (0)	108 (100)
GSE198073	10 (8)	0 (0)
Histotype		
HGSOC	40 (31)	38 (35)
SOC	24 (18)	1 (0.9)
EOC	8 (6.2)	20 (19)
CCOC	6 (4.6)	25 (23)
MOC	6 (4.6)	11 (10)
GCOC	2 (1.5)	0 (0)
LGSOC	0 (0)	1 (0.9)
MXOC	0 (0)	12 (11)
Unspecified	44 (34)	0 (0)
Chemotherapy response		
Resistant	NA	9 (8)
Sensitive	NA	19 (16)
Unknown	NA	80 (68)

Abbreviations: CCOC, Clear Cell ovarian cancer; EOC, Endometrioid ovarian cancer; GCOC, germ cell ovarian cancer; HGSOC, high‐grade serous ovarian cancer; LGSOC, low grade serous ovarian cancer; MOC, Mucinous ovarian cancer; MXOC, Mixed histologies; NA, Not Available; SOC, serous ovarian cancer.

### 2.2 Computational Analysis

**FIGURE 1:**
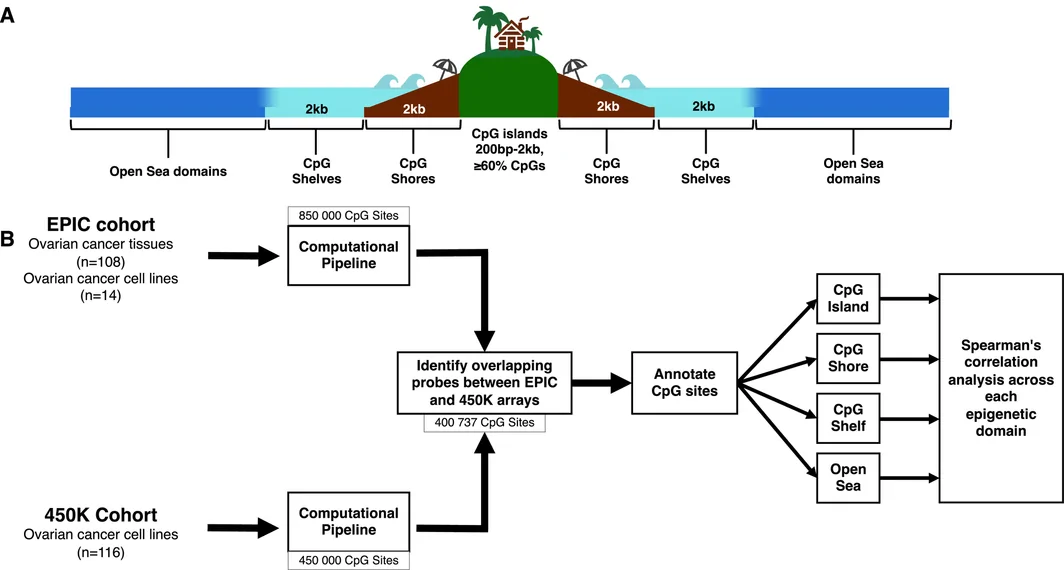
Study design. (A) Epigenetic domains breakdown into CpG islands and proximity to shores, shelves and open sea. (B) The pipeline for computational analysis of publicly available ovarian cancer Illumina DNA methylation array datasets. Following preprocessing, ComBat batch correction was applied to minimise technical variation associated with differences in methylation array platform (450 K versus EPIC) and dataset source prior to comparative analysis.

EPIC and 450 K array data were processed independently using the R package *RnBeads*, version 2.24.0 [27] computational pipeline and annotated to hg19/GRCh37. Briefly, raw EPIC array data (.idat files) were imported, and sample quality was assessed. Data were normalised via SWAN [28], and probes were filtered to remove cross‐reactive, poor performing ones that covered SNPs within their last 5 bases, or were non‐CpG probes. Batch correction was performed using ComBat, an empirical Bayes method implemented within the sva package, to adjust for dataset‐associated variation while retaining biological differences between samples [29]. Analyses were restricted to CpG probes with matching Illumina probe identifiers present on both the EPIC and 450 K arrays [30]. Probes that were removed, redesigned, or assigned different identifiers between array versions were excluded to ensure direct comparison of equivalent CpG sites. Probes were annotated using the EPIC array manifest (MethylationEPIC_v‐1‐0_B2) [31] to Epigenetic Domains: CpG Islands, CpG Shores, CpG Shelves and Open Sea regions (Figure 1A). Genetic domains were classified by the UCSC reference gene group within the Illumina EPIC array manifest file. 5′ regulatory regions encompassed transcriptional start sites, 5′UTRs and 1st exon loci; intragenic regions included exons and 3′UTRs; and loci without any association with genes were classed as intergenic. Enhancers were defined by the Fantom5, Fantom4 and 450 K enhancers within the EPICv1 manifest (MethylationEPIC_v‐1‐0_B2) [31].

### 2.3 Correlation Analysis

The β‐values of CpG sites within each different epigenetic, genetic and enhancer domain were compared between cell lines (*n* = 130) and tissues (*n* = 108). Spearman correlation (r_s_) analysis was conducted due to the non‐normal distribution of the data (Kolmogorov–Smirnov test). Strong correlation was deemed to be median r_s_ ≥ 0.7 (Figure 1B). Boxplots were then generated using the *ggplot2* package, version 3.5.2 [32]. Using the pairwise Wilcoxon test, we examined whether there were differences in the correlation in DNA methylation, firstly within CpG islands, shores, shelves and open sea domains between all cell lines and tissues and subsequently between specific cell line histotypes and their corresponding tissues. Significant differences were determined using adjusted (Benjamini–Hochberg method) *p*‐value ≤ 0.05. Highly significant differences had an adjusted *p*‐value threshold of ≤ 0.01. Effect size (r_effect_) was also measured to allow for accurate interpretation of *p*‐values and understand the degree of true biological differences.

### 2.4 Classification of Suitability of Cell Lines as DNA Methylation Models

Classification thresholds (r_s_ ≥ 0.70, strong concordance; r_s_ ≥ 0.60, moderate‐to‐strong concordance) were selected to provide a consistent framework for comparative evaluation of methylation model suitability. Greater weight was given to CpG‐dense regulatory regions given their role in transcriptional regulation [33]. Based on this framework, cell lines were categorised into four groups based on their suitability as DNA methylation models: (1) ‘Excellent’ and (2) ‘Good’ models had average median r_s_ values across all epigenetic domains of ≤ 0.70 and ≥ 0.60 respectively, for their corresponding histotypes, (3) ‘Acceptable’ models showed average median r_s_ values ≥ 0.60, but individual correlations < 0.70 in CpG islands and/or < 0.50 in CpG shelves and/or open sea domains, and (4) ‘Not recommended for use’ models failed to meet these thresholds (Table S3).

## 3 Results

### 3.1 DNA Methylation Concordance Between Ovarian Cancer Cell Lines and Tissues Is Highest in CpG‐Dense Regions

Before this study, it was unclear how closely DNA methylation patterns in OvCa cell lines reflect those of their tissues of origin. Moreover, it was unknown whether any correlation observed would be consistent across the genome or stronger in specific regions, such as functional elements like CpG islands. So, to address this knowledge gap, we first examined how well DNA methylation correlated between all of the cell lines and tissues at discrete genomic and epigenomic domains. We also examined effect sizes in conjunction with Spearman correlation and *p*‐value to ascertain the magnitude of biological difference in conjunction with statistical significance.

**FIGURE 2:**
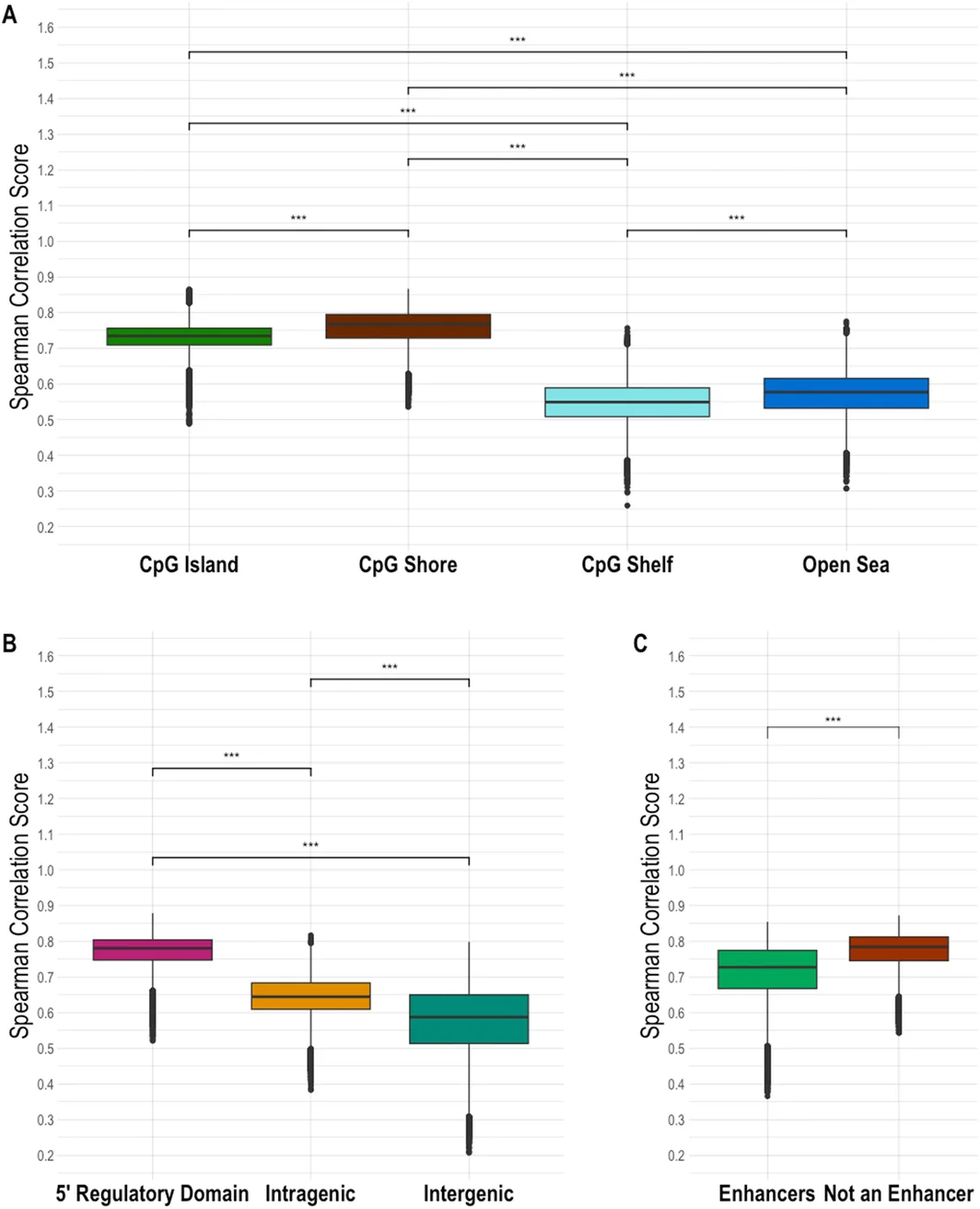
Correlation in DNA methylation between OvCa cell lines and tissues across different genetic and epigenomic domains. Box and whisker illustrate interquartile ranges of median spearman correlation values across CpG sites residing within (A) epigenetic, (B) genetic, and (C) enhancer domains between cell lines (*n* = 130) and tissues (*n* = 108). Adjusted *p*‐value (Benjamini–Hochberg Procedure) significance is shown.

We found a notable trend in how DNA methylation correlated between OvCa cell lines and tissues. Most markedly, CpG dense regions (islands and shores) showed significantly higher correlation levels than CpG sparse regions (CpG shelves or open sea domains) with large effect sizes (r_effect_ ≥ 0.8) (Figure 2A; Table S4). Although the median Spearman correlation (r_s_) of CpG islands (r_s_ = 0.73) was significantly lower than that of shores (r_s_ = 0.76), the effect size (r_effect_ = 0.309) was moderate, indicating that the biological differences are slight. Similarly, the correlation scores between CpG shelves (r_s_ = 0.55) and open sea domains (r_s_ = 0.58) also differed significantly, with a weak effect size (r_effect_ = 0.199), indicative of smaller practical differences. In addition, the correlation scores for CpG shelves and open sea domains exhibited a broader interquartile range than those in islands and shores.

Next, we examined the correlation between genetic domains and also *in cis* enhancer regulatory elements (Figure 2B,C). The correlation in DNA methylation between cell lines and tissues was significantly stronger in 5′ regulatory domains (r_s_ = 0.78) compared to both intragenic (r_s_ = 0.64) and intergenic (r_s_ = 0.59) regions with large effect sizes (r_effect_ ≥ 0.78, adjusted *p*‐value < 0.0001). The correlation coefficients in intergenic regions had the broadest IQR compared to all genetic features. Enhancer domains showed a weaker correlation (r_s_ = 0.73) than regions of the genome not associated with enhancer (r_s_ = 0.78, r_effect_ = 0.423, adjusted *p*‐value < 0.001). So in essence, these data show that DNA methylation in ovarian cancer cell lines and tissues correlate most strongly at areas of the genome that are dense in CpG sites, namely islands and shores, which are typically found at the 5′ regulatory regions of genes.

To address the potential influence of array platform, we performed a within‐platform validation using the subset of cell lines (*n* = 14) also profiled on the EPIC array, repeating the correlation analysis using (a) probes shared between the EPIC and 450 K arrays and (b) the complete EPIC probe set. Both analyses reproduced our original findings, with CpG‐dense domains (islands and shores) showing higher correlations between cell lines and tumour tissues than CpG‐sparse domains (shelves and open sea) (Figure S1).

### 3.2 The Correlation of DNA Methylation Patterns Between Ovarian Cancer Cell Lines and Tissues Is Histotype‐Dependent

**FIGURE 3:**
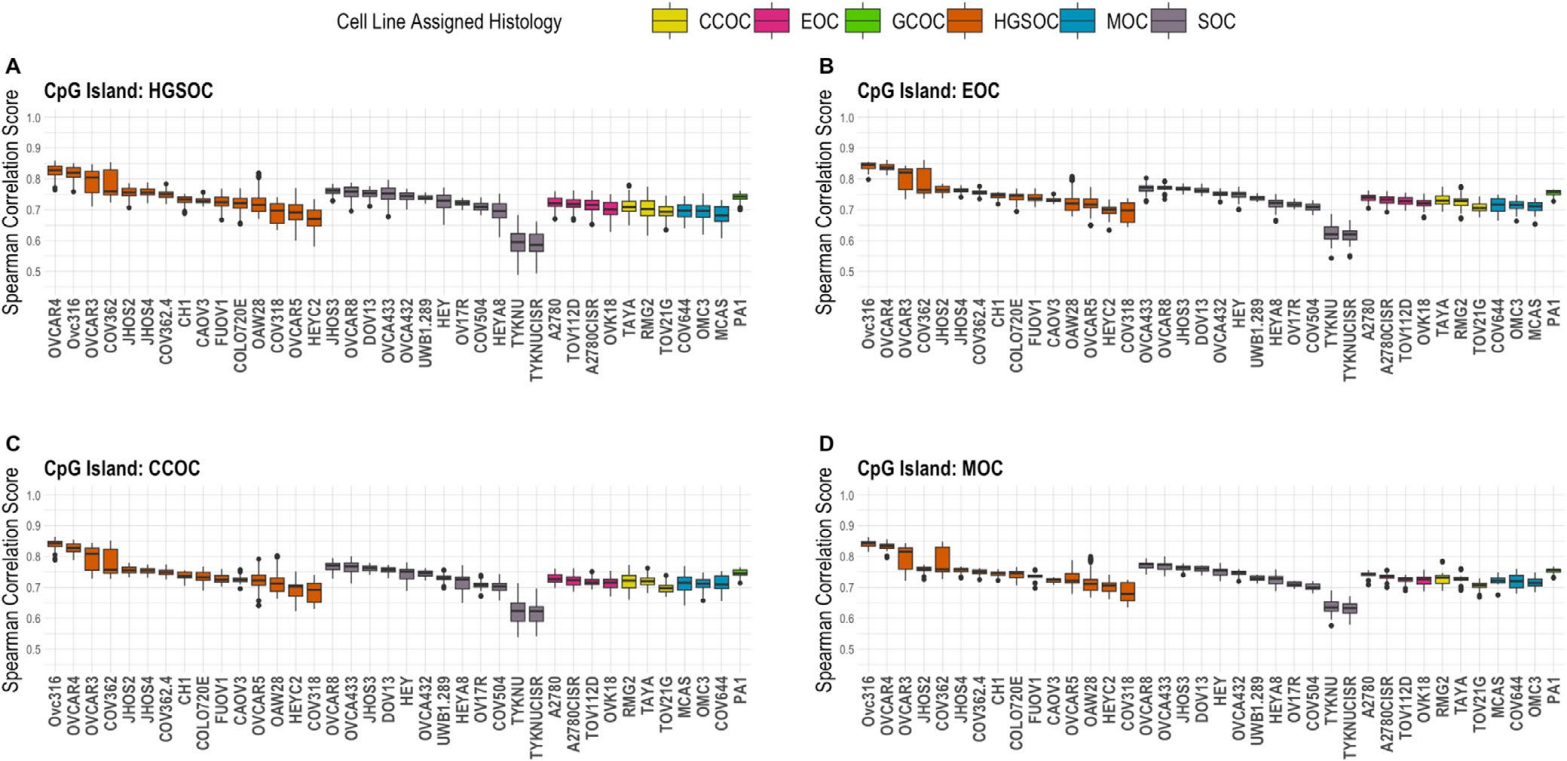
Spearman correlation analysis between cell line DNA methylation at CpG islands and four OvCa tissue histotypes. Box and whisker plots illustrate the interquartile ranges and median spearman correlation value of CpG island methylation for cell lines with specified histotypes compared to (A) High‐grade serous ovarian cancer (HGSOC), (B) Endometrioid ovarian carcinoma (EOC), (C) Clear Cell ovarian carcinoma (CCOC) and (D) Mucinous ovarian carcinoma (MOC) tissues. Cell lines are grouped on the x‐axis according to their assigned histology and arranged by median correlation scores in descending order.

We next assessed how well each OvCa cell line, stratified by designated histotype (shown in Table S2), correlated with its corresponding OvCa tissue of origin. The 130 cell lines were categorised into six different histotypes (Table 1). Overall, CpG islands and shores in each cell line showed strong concordance with tumour samples. The HGSOC cell lines (*n* = 15) exhibited the strongest correlation with OvCa tissues, independent of tissue histology, with median correlation scores ranging from 0.67–0.83 at CpG islands (Figure 3). OVCAR4 showed the highest correlation to HGSOC tissues (median r_s_ = 0.828). Although TYKnu and its daughter cell line TYKnuCisR are often used in HGSOC studies, we classified them as SOC, due to inconsistencies on their grade in the literature. These two cell lines had the lowest correlation to HGSOC tissues (r_s_ = 0.59 and 0.61, respectively; Figure 3A), and indeed to any other OvCa histotype. Of the 15 HGSOC cell lines, HEYC2 demonstrated the poorest correlation to HGSOC tissues, with an r_s_ = 0.67, whereas Ovc316 correlated best to EOC, CCOC and MOC histotypes, with r_s_ of 0.85, 0.84 and 0.84, respectively.

All four EOC cell lines (A2780, A270CisR, TOV112D and OVK18) correlated well to the EOC tissues with median r_s_ ≥ 0.7 in both CpG islands and shores (Figure 3B, Figure S2). The three CCOC cell lines (RMG2, TAYA and TOV21G) also showed strong correlation to their respective tissues in CpG islands, with median r_s_ ranging from 0.70 to 0.72 (Figure 3C). The three MOC cell lines performed marginally better, with median correlation values of 0.72, 0.72 and 0.71 for MCAS, COV644 and OMC3, respectively (Figure 3D). Correlation at CpG shores was also strong for each cell line with their corresponding tissue of origin (median r_s_ ≥ 0.72).

Consistently, the correlation in DNA methylation between discrete cell line histotypes and their corresponding tissues was lower overall for the CpG‐sparse open sea domains, with all r_s_ ranging from 0.42 to 0.67. The highest correlation coefficient within open sea domains was 0.67 between the SOC cell line COV504 and HGSOC tissues. This cell line also had the highest r_s_ (0.67) to EOC tissues for the same epigenetic domain (Figure S4A,B). OVCA432, a SOC line, had the strongest correlation to CCOC and MOC tissues, with median r_s_ of 0.64 and 0.66 respectively (Figure S4C,D). This decrease in correlation from CpG dense loci (CpG islands Figure 3, and shores Figure S2), to CpG sparse areas of the genome (shelves Figure S3, and open sea domains Figure S4) holds true across the majority of cell lines in this study.

### 3.3 Unspecified and Ungraded Serous Ovarian Cancer Cell Lines Show the Strongest Correlation With Mixed Ovarian Cancer Tissues

The data show distinct similarities and differences in DNA methylation across CpG dense and sparse areas of the genome between cell lines to their corresponding tissues. The same trend was observed when comparing cell lines to MXOC tissues (Figure S5). While rare, some patients present clinically with two or more histotypes [34]. In this dataset, many of the mixed tissues contained the serous pathology [25]. Notably SOC cell lines maintained a higher correlation to MXOC tissues across each domain. Of the SOC cell lines, JHOS‐3 had the highest correlation to MXOC tissues, specifically in CpG shores (median r_s_ = 0.80) (Figure S5A,B). COV504 and OVCA432 had the strongest correlation (r_s_ ≥ 0.62) to shelf and open sea domains compared to all other cell lines (Figure S5C,D).

**FIGURE 4:**
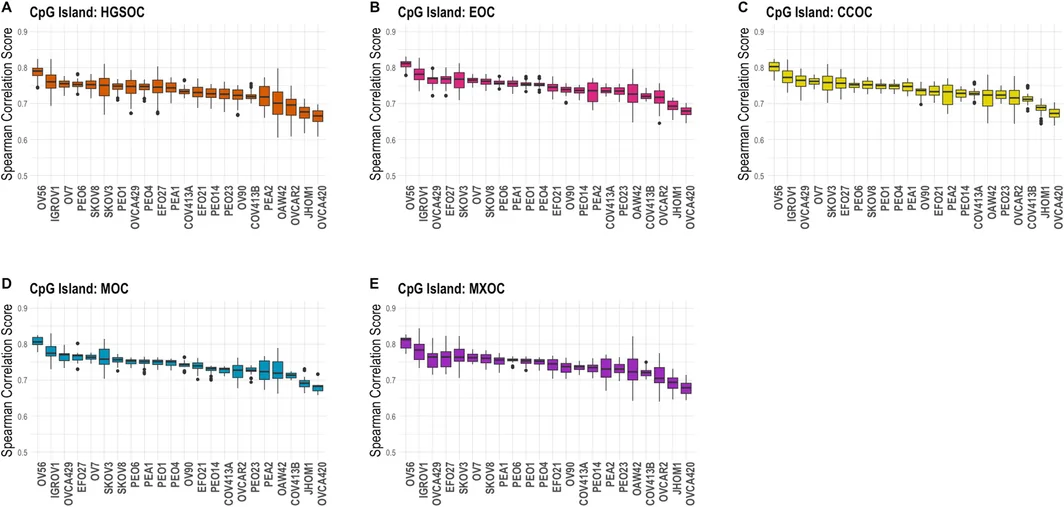
Spearman correlation analysis of CpG island DNA methylation between unspecified cell lines and five OvCa tissue histotypes. Box and whisker illustrate the interquartile ranges and median spearman correlation value of each cell line derived from unspecified histotype origin compared to either (A) High‐grade serous ovarian cancer (HGSOC), (B) Endometrioid ovarian carcinoma (EOC), (C) Clear Cell ovarian carcinoma (CCOC) (D) Mucinous ovarian carcinoma (MOC), (E) Mixed ovarian cancer (MXOC) tissue samples. Cell lines are organised in descending order on the x‐axis according to median correlation scores against each indicated ovarian cancer tissue histotype.

Twenty‐two cell lines were not assigned a histology, due to lack of information from the prementioned sources or due to conflicting reports (‘unspecified cell lines’) (Table S2). These demonstrated strong median r_s_ values (0.67–0.81) to all OvCa histotypes within CpG islands (Figure S5). We note the same pattern as before, where this correlation increases marginally in CpG shores for the majority of cell lines, but notably is weaker as we progress to CpG shelves and open sea domains (Figure S6). These unspecified lines have a moderate, but stronger r_s_ within open sea domains compared to cell lines with specified histology (r_s_ range 0.48–0.70). OV56 correlated best with MXOC OvCa tissues within CpG islands r_s_ = 0.81 and maintained a strong correlation to MXOC tissues as we progress to CpG shores r_s_ = 0.84, which weakened in CpG shelves r_s_ = 0.64 and open sea domains r_s_ = 0.66. The unspecified cell line, OV56, is notable as it correlated best to all tissue types (r_s_ ≥ 0.8 in CpG islands and shores). Of the unspecified cell lines, OVCA420 and JHOM1 consistently correlated weakest to all tissues across CpG islands with a correlation median < 0.70 in OvCa tissues (Figure 4).

### 3.4 Recommendations for the Use of OvCa Cell Lines in DNA Methylation Studies

Finally, we integrated our methylation analysis with previous molecular profiling studies of ovarian cancer cell lines, including genomic and transcriptomic characterisation [8, 9, 10, 11, 12, 23, 24] to provide guidance on the suitability of OvCa cell lines for DNA methylation studies (Table 2). These recommendations are intended to identify models that best preserve histotype‐associated methylation patterns and should not be interpreted as definitive histotype reassignment. Cell lines were categorised into four categories based on their suitability as DNA methylation models, ‘excellent’, ‘good’, ‘acceptable’ or ‘not recommended for use’ based on average median r_s_ values across all epigenetic domains for their corresponding histotypes (Table S3).

**TABLE 2:** Recommended ovarian cancer cell lines for DNA methylation studies by histotype.

Cell Line	Assigned Histotype^a^	Recommendations for use in DNA methylation studies^b^		
RMG2	CCOC	Good CCOC model		
TOV21G	CCOC	Acceptable CCOC model		
TAYA	CCOC	Not recommended for use		
TOV112D	EOC	Good EOC model		
A2780	EOC	Good EOC model		
A2780CISR	EOC	Good EOC model		
OVK18	EOC	Acceptable EOC model		
COV644	MOC	Good MOC model		
MCAS	MOC	Good MOC model		
OMC3	MOC	Good MOC model		
JHOS4	HGSOC	**Excellent HGSOC model**		
OVCAR4	HGSOC	**Excellent HGSOC model**		
COV362.4	HGSOC	Good HGSOC model		
OVCAR3	HGSOC	Good HGSOC model		
COV362	HGSOC	Good HGSOC model		
Ovc316	HGSOC	Good HGSOC model		
OAW28	HGSOC	Good HGSOC model		
JHOS2	HGSOC	Good HGSOC model		
CH1	HGSOC	Good HGSOC model		
CAOV3	HGSOC	Good HGSOC model		
FUOV1	HGSOC	Good HGSOC model		
COLO720E	HGSOC	Good HGSOC model		
COV318	HGSOC	Acceptable HGSOC model		
OVCAR5	HGSOC	Not recommended for use		
HEYC2	HGSOC	Not recommended for use		
COV504	SOC	**Excellent HGSOC model**		
OVCA432	SOC	**Excellent HGSOC model**		
OV17R	SOC	Requires further integrated evaluation before LGSOC modelling		
UWB1.289	SOC	Requires further integrated evaluation before LGSOC modelling		
OVCA433	SOC	Requires further integrated evaluation before LGSOC modelling		
JHOS3	SOC	Requires further integrated evaluation before LGSOC modelling		
OVCAR8	SOC	Requires further integrated evaluation before LGSOC modelling		
DOV13	SOC	Requires further integrated evaluation before LGSOC modelling		
HEY	SOC	Requires further integrated evaluation before LGSOC modelling		
HEYA8	SOC	Requires further integrated evaluation before LGSOC modelling		
TYKNUCISR	SOC	Not recommended for use		
TYKNU	SOC	Not recommended for use		
PA1	GCOC	Need to study against GCOC tissue		
OV56	Unspecified	**Excellent General OvCa or MXOC model**		
PEO6	Unspecified	**Excellent General OvCa or MXOC model**		
PEO4	Unspecified	**Excellent HGSOC model**		
PEO1	Unspecified	**Excellent HGSOC model**		
COV413A	Unspecified	**Excellent HGSOC model**		
PEA2	Unspecified	Not recommended for use		
PEO23	Unspecified	Not recommended for use		
SKOV3	Unspecified	Not recommended for use		
OV90	Unspecified	Not recommended for use		
OVCA420	Unspecified	Not recommended for use		
OVCA429	Unspecified	Not recommended for use		
IGROV1	Unspecified	Not recommended for use		
COV413B	Unspecified	Not recommended for use		
OAW42	Unspecified	Not recommended for use		
JHOM1	Unspecified	Not recommended for use		
EFO27	Unspecified	Not recommended for use		
EFO21	Unspecified	Not recommended for use		
OV7	Unspecified	Not recommended for use		
OVCAR2	Unspecified	Not recommended for use		
SKOV8	Unspecified	Not recommended for use		
PEA1	Unspecified	Not recommended for use		
PEO14	Unspecified	Not recommended for use		
Traffic light system	**Excellent Model**	Good Model	Acceptable model	Not recommended

*Note:* Cell lines were categorised into four categories based on their suitability as DNA methylation models, ‘excellent’, ‘good’, ‘acceptable’ or ‘not recommended for use’. ‘Excellent’ and ‘good’ DNA methylation cell line models achieved average median r_s_ values of ≥ 0.70 and ≥ 0.60, respectively, across all epigenetic domains for their corresponding histotypes. ‘Acceptable’ DNA methylation cell line models exhibited an average median r_s_ ≥ 0.60, however individual correlation in CpG islands and/or shores fell below an r_s_ of 0.70, and/or below 0.50 in CpG shelves and/or open sea domains. Cell line models that were ‘Not recommended for use’ achieved an average median r_s_ ≤ 0.60 across all epigenetic domains.

^a^ Assigned histotypes were derived from meta‐analysis of multiple sources, see Table S2.

^b^ Correlation scores for each cell line and tissue are presented in Table S3.

Using this approach, RMG2 was deemed to be a good DNA methylation model of the CCOC histotype, while TOV21G was deemed acceptable, with a median r_s_ ≤ 0.7 in CpG islands. In contrast, TAYA is not recommended for use as a CCOC cell line model, with an average median r_s_ ≤ 0.60 across all epigenetic domains. For the EOC histotype, A2780, A2780CISR and TOV112D were all deemed good DNA methylation models, with OVK18 being classified as acceptable. OVK18 correlated strongly in CpG dense areas of the genome (median r_s_ ≥ 0.70) yet poorly in open sea domains (median r_s_ ≤ 0.48). All three MOC cell lines (COV644, MCAS and OMC3) were deemed to be good models for DNA methylation studies in this histotype, with an average median r_s_ ≥ 0.63 across all epigenetic domains. As the most common and studied histotype, it is not surprising that we identified multiple good HGSOC cell line models (Table 2, Table S3). We identified both JHOS‐4 and OVCAR4 as excellent, highly representative HGSOC DNA methylation models with an average median r_s_ ≥ 0.72. In contrast, COV318 was deemed acceptable, while HEYC2 and OVCAR5 are not recommended for use in DNA methylation studies due to poor correlation across all domains (HEYC2) and OVCAR5 previously identified as gastrointestinal in origin [35].

Interestingly, three unspecified cell lines (PEO1, PEO4 and COV413A) and two SOC cell lines (COV504 and Cov432) showed high concordance to HGSOC tissues, with correlation scores (average median r_s_ ≥ 0.70), comparable to JHOS‐4 and OVCAR4. Accordingly, these cell lines were classified as excellent HGSOC DNA methylation models. OV56 and PEO6 were excellent DNA methylation models across all OvCa histotypes, including MXOC, achieving a median r_s_ ≥ 0.7. In contrast, TKNUCISR and TKNU performed poorest in this analysis and are not recommended for use in OvCa DNA methylation studies. Although other SOC lines showed comparable methylation correlations to known HGSOC models, their classification remains uncertain without direct comparison to LGSOC tissues. Several cell lines also contain genomic alterations that may be inconsistent with classical LGSOC biology [10, 12]. Therefore, while these models demonstrated methylation similarity to available LGSOC‐related profiles, we recommend that OV17R, UWB1.289, OVCA433, JHOS3, OVCAR8, DOV13, HEY and HEYA8 undergo further integrated genomic, transcriptomic and methylation assessment before being considered representative LGSOC models.

## 4 Discussion

Here, we present the first genome‐wide assessment of how representative OvCa cell lines are of their corresponding tissue histotypes at the level of DNA methylation. This is an important question because the molecular biology, diagnostic stage, therapeutic response, prognosis and origin of OvCa are all heavily influenced by histotype [6], yet, many OvCa cell lines lack detailed histotype annotation. Where such information is available, studies have sometimes found molecular dissimilarities between cell lines and their tissue of origin. Understanding how representative cell lines are of their in vivo tissue type is therefore fundamental: without this knowledge, experimental findings risk misdirecting research, generating unrepresentative results and undermining in vivo reproducibility, ultimately wasting valuable research resources. A previous study demonstrated distinguishability between OvCa histotypes based on promoter hypermethylation at 41 genes. However, LGSOC and HGSOC were considered as a single subtype [36]. Our study is the first to report how relevant and representative OvCa cell lines are as DNA methylation models of their respective histotypes using an unbiased genome‐wide approach.

A strong and consistent trend emerged across the dataset, irrespective of cell line or histotype: CpG‐dense regions (islands and shores) showed the highest correlation between OvCa cell lines and tissues, while CpG‐sparse regions (shelves and open sea) showed weaker, more variable correlation. This was not attributable to differences in probe representation across domains (CpG islands: 127,527 probes, 31.83%; shores: 94,727, 23.64%; shelves: 38,264, 9.55%; open sea: 140,111, 34.97%), as open sea regions contained the highest proportion of analysed probes yet still showed reduced correlation relative to CpG islands and shores. A plausible contributing factor is that CpG density influences methylation conservation as cells adapt to culture: neighbouring CpG sites are known to share methylation status through ‘co‐methylation’, which is strongest between closely‐spaced sites and decreases as inter‐site distance increases, in a non‐stochastic manner [37, 38]. This is consistent with a model in which densely‐spaced CpG sites ‘collaborate’ to maintain methylation states through cell division, recruiting enzymes that reinforce the existing methylated or unmethylated state [39, 40]. This mechanism may help explain the broad distinction between CpG‐dense and CpG‐sparse domains, although it does not fully account for finer‐grained differences within CpG‐dense regions: notably, we observed greater conservation within CpG shores than within CpG islands, despite islands being the denser of the two features. This suggests that factors beyond local CpG density also shape methylation conservation in cell line models. Studies typically focus on CpG islands, potentially overlooking the functional importance of other epigenetic domains. For example, tissue‐specific gene expression has been shown to be driven by DNA methylation patterns at CpG shores more strongly than CpG islands [33]. The stronger conservation of shore methylation may therefore reflect the particular importance of this domain in maintaining cellular identity, potentially outweighing the contribution of density‐driven co‐methylation within CpG islands. Further work will be needed to clarify the relative contributions of CpG density and region‐specific regulatory function to methylation conservation in vitro.

Enhancers are among the most dynamic genomic elements, particularly in terms of DNA methylation [41]. This adaptability is known as enhancer reprogramming and is critical to cancer progression [42], contributing to chemoresistance [43]. Epigenetic reprogramming more broadly also supports cellular adaptation to the in vitro environment [44]. The reduced conservation of enhancer methylation observed between ovarian cancer cell lines and tumour tissues may therefore reflect adaptation to culture conditions; enhancer activity is highly responsive to external signals, and the absence of tumour microenvironmental components, including stromal and immune‐derived cues, in standard two‐dimensional cell culture may contribute to differences in enhancer methylation between cell lines and primary tumours [42]. Together, these factors may explain the greater variability observed at enhancer regions relative to CpG islands, shores and promoter‐associated regions.

Across all five OvCa histologies investigated, HGSOC lines showed the strongest correlation to tissues, across all histotypes in CpG islands. Since cell lines must retain key cancer hallmarks, such as sustained proliferation, resistance to cell death and altered metabolism to survive in culture [45], and HGSOC is the most malignant histotype, it is conceivable that HGSOC‐associated methylation profiles are shared with other histotypes to some extent. Notably, some SOC cell lines correlated more strongly with HGSOC tissue than several standard HGSOC cell lines themselves. This may partly reflect historical classification, as SOC was only formally divided into HGSOC and LGSOC by the WHO in 2014 [6]. However, methylation similarity alone cannot establish histotype identity: these SOC cell lines should be interpreted alongside other molecular features, including *TP53* status, *BRCA* alterations, copy number changes and transcriptomic profiles, as discordance between omic layers is expected given that each captures distinct aspects of tumour biology. We therefore regard these cell lines as candidates for methylation‐focused studies rather than as definitively reclassified models, particularly since LGSOC is a comparatively indolent disease with a better prognosis than HGSOC [46], and confirmation against LGSOC tissue is warranted in future work.

Some of our findings diverged from previous molecular characterisation studies. McCabe et al. carried out transcriptomic profiling of OvCa cell lines and found that A2780 clustered separately from established models, suggesting a possible non‐ovarian origin. In contrast, we found that the DNA methylation profile of A2780 and its daughter line A2780CisR correlated well with EOC. A2780 was derived from an African American patient and has been reported to have 91.08% African genomic ancestry [23], raising the possibility that patient ancestry contributes to the molecular differences observed between studies; however, ancestry data were unavailable for our tissue cohort and were not reported by McCabe et al. so its contribution remains unclear. This gap underscores the need for greater diversity in cancer omic datasets. More broadly, these findings show that concordance at one molecular layer does not necessarily reflect broader biological similarity, as DNA methylation, transcriptomic, genomic and functional profiles capture distinct aspects of tumour biology and may be discordant between model systems. A2780 may therefore be a suitable model for EOC methylation‐focused studies, but additional caution is warranted when using it for transcriptomic, functional, or therapeutic response studies. More generally, cell line selection should be guided by the specific biological question and, where possible, supported by integrated molecular evidence. Indeed, some ovarian cancer cell lines have undergone histotype reclassification as additional genomic and molecular data have become available, underscoring the need to continually integrate emerging evidence when selecting models. Our recommendations therefore reflect model suitability based on currently available evidence, rather than fixed biological classifications.

This study has several limitations. The tumour reference cohort was derived from a publicly available methylation dataset, and we were therefore reliant on the original histotype annotations, which were based on morphological assessment of H&E‐stained sections without additional immunohistochemical or molecular data. This is an important limitation, as many of the ovarian cancer histotypes display overlapping morphological features, and so ancillary testing is often required for definitive classification [6]. Another, albeit related issue, is that publicly available methylation data for non‐HGSOC histotypes remain limited. In this case, this resulted in unequal representation within the reference cohort (including only a single LGSOC and SOC tumour). Correlations involving these rare histotypes should therefore be interpreted cautiously pending validation in larger, independently characterised cohorts with comprehensive pathological, immunohistochemical and molecular profiling. A further limitation is the integration of methylation datasets using different Illumina platforms, namely the 450 K and EPIC array. Although these were processed using a common pipeline restricted to overlapping probes and corrected with ComBat, residual platform effects cannot be fully excluded. However, the consistent genomic domain‐specific pattern observed across annotations, greater concordance in CpG‐dense regulatory regions and reduced concordance in CpG‐sparse regions, despite Open Sea regions containing the highest proportion of analysed probes, suggests that this pattern likely reflects genuine biological differences in methylation conservation rather than technical artefact. It is also important to note that the thresholds we used to classify OvCa cell lines for methylation studies represent an empirical framework [47] for prioritising models by methylation similarity rather than absolute biological cut‐offs; future studies incorporating additional molecular and functional measures will be needed to determine whether methylation concordance predicts broader model fidelity.

Most research has moved beyond a one‐size‐fits‐all approach, increasingly recognising each ovarian cancer histotype as a distinct disease [5]. However, some studies still group epithelial ovarian cancers as a single entity without histotype classification. This trend is also evident in recent clinical trials, which do not define epithelial ovarian cancer histotypes, but also combine them with fallopian tube and primary peritoneal cancers [48]. If a treatment is effective, regardless of histotype, this could be incredibly valuable. Therefore, it is essential to maintain cell line models that represent individual histotypes as well as those with broader applicability. To address this need for histotype‐independent ovarian cancer DNA methylation research, we recommend OV56 and PEO6, which showed robust methylation concordance across the histotypes examined here. Confidence in this conclusion is more limited for LGSOC and SOC in particular, given the small number of reference tumours available for these subtypes. Nonetheless, these models offer a practical starting point for histotype‐independent methylation research, alongside the histotype‐specific recommendations detailed.

## 5 Conclusion

Overall, our findings reveal a genomic domain‐specific gradient of methylation conservation between OvCa cell lines and tumour tissue, with greater fidelity in CpG‐dense regulatory regions and greater divergence in CpG‐sparse and enhancer regions. Model suitability was also histotype‐dependent, and we provide specific recommendations accordingly, including OV56 and PEO6 as candidates for histotype‐independent methylation studies. These findings underscore that a one‐size‐fits‐all approach to cell line selection is often inappropriate for studying OvCa biology in vivo. We recommend that future cell line selection for DNA methylation research be guided by both genomic context and histotype‐specific model performance.

## Author Contributions

**Carson Stacy:** methodology, visualization, writing – review and editing, formal analysis, data curation. **Asia Jordan:** conceptualization, investigation, funding acquisition, writing – original draft, methodology, validation, visualization, writing – review and editing, formal analysis, project administration. **Antoinette S. Perry:** supervision, writing – review and editing, funding acquisition. **Tuan Zea Tan:** methodology, resources, writing – review and editing. **Sudipto Das:** supervision, writing – review and editing. **Abby Caffrey:** investigation. **Kellie Dean:** supervision, writing – review and editing. **Aideen McCabe:** methodology, investigation, formal analysis. **Ruby Yun‐Ju Huang:** resources, methodology, writing – review and editing.

## Funding

This work was funded by the Irish Research Council and Breakthrough Cancer Research through the Enterprise Partnership Scheme (EPSPG/2021/169).

## Ethics Statement

The authors have nothing to report.

## Consent

The authors have nothing to report.

## Conflicts of Interest

The authors declare no conflicts of interest.

## Supporting information


**Figure S1:** Correlation in DNA methylation between OvCa cell lines (*n* = 14) and tissues (108) within the EPIC array dataset across epigenomic domains. Box and whisker illustrate interquartile ranges of median spearman correlation values across CpG sites residing within epigenetic domains. (A) the subset of probes on the 850 K which overlap between the 450 K array, (B) the correlation across the entire EPIC array dataset. Adjusted *p*‐value (Benjamini–Hochberg Procedure) significance is shown.
**Figure S2:** Spearman correlation analysis between cell line DNA methylation at CpG Shores and four OvCa tissue histotypes. Box and whisker illustrate the interquartile ranges and median spearman correlation value of methylation at CpG shores for cell lines with specified histotypes compared to either (A) High‐grade serous ovarian cancer (HGSOC), (B) Endometrioid ovarian carcinoma (EOC), (C) Clear Cell ovarian carcinoma (CCOC) (D) Mucinous ovarian carcinoma (MOC) tissues. Cell lines are organised by correlation scores arranged in descending order within each assigned histology. The assigned histology of each cell line is indicated by the key.
**Figure S3:** Spearman correlation analysis between cell line DNA methylation at CpG shelves and four OvCa tissue histotypes. Box and whisker illustrate the interquartile ranges and median spearman correlation value of methylation at CpG shelves for cell lines with specified histotypes compared to either (A) High‐grade serous ovarian cancer (HGSOC), (B) Endometrioid ovarian carcinoma (EOC), (C) Clear Cell ovarian carcinoma (CCOC) (D) Mucinous ovarian carcinoma (MOC) tissues. Cell lines are organised by correlation scores arranged in descending order within each assigned histology. The assigned histology of each cell line is indicated by the key.
**Figure S4:** Spearman correlation analysis between cell line DNA methylation at Open Sea domains and four OvCa tissue histotypes. Box and whisker plots illustrate the interquartile ranges and median spearman correlation value of methylation in open sea domains for cell lines with specified histotypes compared to either (A) High‐grade serous ovarian cancer (HGSOC), (B) Endometrioid ovarian carcinoma (EOC), (C) Clear Cell ovarian carcinoma (CCOC) and (D) Mucinous ovarian carcinoma (MOC) tissue samples and cell lines. Cell lines are grouped on the x‐axis according to their assigned histology and arranged in descending order by median correlation scores. The assigned histology of each cell line is indicated by the key.
**Figure S5:** Spearman correlation analysis between cell line DNA methylation compared to mixed OvCa tissue (MXOC) in each epigenetic domain. Boxplots illustrate the interquartile ranges and median spearman correlation value of methylation between cell lines with specified histotypes compared to mixed ovarian histotype tissue (MXOC) within (A) CpG islands, (B) CpG shores, (C) CpG shelves and (D) Open Sea domains. Cell lines are organised on the x‐axis based on median correlation scores in descending order within each assigned histology. The assigned histology of each cell line is indicated by the key.
**Figure S6:** Spearman correlation analysis DNA methylation between unspecified cell lines and five OvCa tissue histotypes. Box and whisker illustrate the interquartile ranges and median spearman correlation scores of each cell line derived from unspecified histotype origin compared to either HGSOC, EOC, CCOC, MOC or MXOC as indicated in the key. DNA methylation is compared across; (A–E) CpG shores, (F–J) CpG Shelves and (K–O) Open Sea domains. For each plot cell lines are organised in descending order on the x‐axis by median correlation score in descending order against each indicated ovarian cancer tissue histotype.

**Table S1:** Source of Illumina DNA methylation array datasets for OvCa cell lines.
**Table S2:** Histotype classifications of OvCa cell lines used in this study and other independent studies.
**Table S3:** Median correlation scores for epigenetic domains between OvCa cell lines and tissue.
**Table S4:** Wilcoxon effect across genetic and epigenetic domains.

## Data Availability

The data that supports the findings of this study are available in the Supporting Information of this article.
