# Supplementary material for: Genome‐Wide Assessment of DNA Methylation Fidelity Identifies Representative Ovarian Cancer Cell Line Models
Source: J Cell Mol Med. 2026 Aug 22;30(16):e71334. doi: 10.1111/jcmm.71334 (PMC13498969; doi:10.1111/jcmm.71334)
Supplement: Supplementary file 1 [file JCMM-30-e71334-s002.docx]

**Supplementary Figure 1. Correlation in DNA methylation between OvCa cell lines (n=14) and tissues (108) within the EPIC array dataset across epigenomic domains.** Box and whisker illustrate interquartile ranges of median spearman correlation values across CpG sites residing within epigenetic domains. **A)** the subset of probes on the 850K which overlap between the 450K array, **B)** the correlation across the entire EPIC array dataset. Adjusted p-value (Benjamini–Hochberg Procedure) significance is shown.

**Supplementary Figure 2 Spearman correlation analysis between cell line DNA methylation at CpG Shores and four OvCa tissue histotypes.** Box and whisker illustrate the interquartile ranges and median spearman correlation value of methylation at CpG shores for cell lines with specified histotypes compared to either **A)** High-grade serous ovarian cancer (HGSOC), **B)** Endometrioid ovarian carcinoma (EOC), **C)** Clear Cell ovarian carcinoma (CCOC) **D)** Mucinous ovarian carcinoma (MOC) tissues. Cell lines are organised by correlation scores arranged in descending order within each assigned histology. The assigned histology of each cell line is indicated by the key.

**Supplementary Figure 3. Spearman correlation analysis between cell line DNA methylation at CpG shelves and four OvCa tissue histotypes.** Box and whisker illustrate the interquartile ranges and median spearman correlation value of methylation at CpG shelves for cell lines with specified histotypes compared to either **A)** High-grade serous ovarian cancer (HGSOC), **B)** Endometrioid ovarian carcinoma (EOC), **C)** Clear Cell ovarian carcinoma (CCOC) **D)** Mucinous ovarian carcinoma (MOC) tissues. Cell lines are organised by correlation scores arranged in descending order within each assigned histology. The assigned histology of each cell line is indicated by the key.

**Supplementary Figure 4. Spearman correlation analysis between cell line DNA methylation at Open Sea domains and four OvCa tissue histotypes.** Box and whisker plots illustrate the interquartile ranges and median spearman correlation value of methylation in open sea domains for cell lines with specified histotypes compared to either **A)** High-grade serous ovarian cancer (HGSOC), **B)** Endometrioid ovarian carcinoma (EOC), **C)** Clear Cell ovarian carcinoma (CCOC) and **D)** Mucinous ovarian carcinoma (MOC) tissue samples and cell lines. Cell lines are grouped on the x-axis according to their assigned histology and arranged in descending order by median correlation scores. The assigned histology of each cell line is indicated by the key.

**Supplementary Figure 5. Spearman correlation analysis between cell line DNA methylation compared to mixed OvCa tissue (MXOC) in each epigenetic domain.** Boxplots illustrate the interquartile ranges and median spearman correlation value of methylation between cell lines with specified histotypes compared to mixed ovarian histotype tissue (MXOC) within **A)** CpG islands, **B)** CpG shores, **C)** CpG shelves and **D)** Open Sea domains. Cell lines are organised on the x-axis based on median correlation scores in descending order within each assigned histology. The assigned histology of each cell line is indicated by the key.

**Supplementary Figure 6. Spearman correlation analysis DNA methylation between unspecified cell lines and five OvCa tissue histotypes.** Box and whisker illustrate the interquartile ranges and median spearman correlation scores of each cell line derived from unspecified histotype origin compared to either HGSOC, EOC, CCOC, MOC or MXOC as indicated in the key. DNA methylation is compared across; **A-E)** CpG shores, **F-J)** CpG Shelves and **K-O)** Open Sea domains. For each plot cell lines are organised in descending order on the x-axis by median correlation score in descending order against each indicated ovarian cancer tissue histotype.
